# Current status and perspectives of interventional clinical trials for brain metastases: analysis of ClinicalTrials.gov

**DOI:** 10.1186/s13014-023-02243-2

**Published:** 2023-04-04

**Authors:** Paolo Tini, Francesco Marampon, Martina Giraffa, Samira Bucelli, Maximilian Niyazi, Claus Belka, Giuseppe Minniti

**Affiliations:** 1 Department of Medicine, Surgery and Neurosciences, University of Siena, 53100 Siena, Italy; 2 Department of Radiological Sciences, Oncology and Anatomical Pathology, Sapienza University of Rome, Rome, Italy; 3 UPMC Hillman Cancer Center, San Pietro Hospital FBF, Rome, Italy; 4 Department of Radiation Oncology, University Hospital, LMU Munich, Munich, Germany; 5 German Cancer Consortium (DKTK), Munich, Germany; 6 Bavarian Cancer Research Center (BZKF), Munich, Germany; 7 IRCCS Neuromed, 86077 Pozzilli, IS Italy

**Keywords:** Brain metastases, Clinical trials, Ongoing trials, Interventional

## Abstract

**Background:**

The management of brain metastases (BM), the major cause of cancer morbidity and mortality, is becoming an emerging area of interest. Surgery, whole brain radiation therapy (WBRT), or stereotactic radiosurgery (SRS), have historically been the main focal treatments for BM. However, the introduction of innovative targeted- and immune-based therapies is progressively changing the paradigm of BM treatment, resulting in an increase in clinical trials investigating new therapeutic strategies.

**Methods:**

Using ClinicalTrials.gov, the largest clinical trial registry with over 400,000 registered trials, we performed an analysis of phase II and phase III ongoing trials evaluating different systemic therapies, radiotherapy (RT), and surgery given alone or in combination in patients with BM.

**Results:**

One hundred sixty-eight trials, 133 phase II and 35 phase III; the largest part having primarily the curative treatment of patients with BM from lung cancer, breast cancer and melanoma, were selected. One hundred sixty-three trials used systemic therapies. One hundred thirteen used tyrosine kinase inhibitors, more frequently Osimertinib, Icotinib and Pyrotinib, 50 used monoclonal antibodies, more frequently Trastuzumab, Pembrolizumab, Nivolumab, 20 used conventional chemotherapies whilst no oncological active drugs were used in 6 trials. Ninety-six trials include RT; 54 as exclusive treatment and 42 in combination with systemic therapies.

**Conclusion:**

Systemic targeted- and/or immune-based therapies, combined or not with RT, are increasingly used in the routine of BM treatment. SRS is progressively replacing WBRT. All these trials intend to address multiple questions on the management of patients with BMs, including the recommended upfront treatment for different cancer histologies and the optimal timing between systemic therapies and radiation regarding brain control and neurocognitive outcome and quality of life.

**Supplementary Information:**

The online version contains supplementary material available at 10.1186/s13014-023-02243-2.

## Introduction

Brain metastases (BM) are tenfold more common than primary malignant brain tumors and represent the most devastating neurologic complications of cancer. Up to 30–40% of cancer patients develop BM with lung, breast, and melanoma cancers resulting the leading cause of BM formation, causing 67–88% of all clinical cases of BM [1–3]. Traditionally BM have been treated with surgical resection, whole brain radiotherapy (WBRT), and stereotactic radiosurgery (SRS) [4]. However, the development of targeted- and immuno-based therapies is revolutionizing the management of BM [5–8].

Targeted—[9–11] and immune-based therapies [12–14] are largely used to treat several cancers, including lung, breast and melanoma cancers. Epidermal growth factor receptor (EGFR) tyrosine kinase Inhibitor (TKI) (gefitinib, erlotinib, afatinib, icotinib, and osimertinib, dacomitinib) and anaplastic lymphoma kinase (ALK) inhibitors (crizotinib, ceritinib, alectinib, brigatinib, and lorlatinib) are used for treating EGFR-mutated or ALK-rearranged non-small-cell lung cancers (NSCLC) patients, respectively [11]. Epidermal growth factor receptor 2 (HER2) inhibitors, including pertuzumab, trastuzumab, trastuzumab-emtansine, trastuzumab deruxtecan, lapatinib, tucatinib, and neratinib are used for treating HER2-positive breast cancer [10], whilst inhibitors of BRAF (dabrafenib, vemurafenib) are given together with MEK inhibitors (trametinib, cometinib) in BRAF-mutated melanoma [9]. At the same manner, immune checkpoint inhibitors (ICI) anti PD-1/PDL-1 (nivolumab, pembrolizumab, atezolizumab, durvalumab) and anti-CTLA-4 inhibitors (Ipilimumab, tremelimumab) are increasingly used for treating patients with advanced disease [12–14], whilst combining targeted- and immune-based therapies is being evaluated in a variety of solid tumors [15]. Notably, according to the ability of these drug to penetrate the blood–brain barriers, targeted- and immune-based therapies, alone or in combination, have shown a therapeutic efficiency in treating BM [5–8], whilst increasing evidence suggest their use in combination with radiotherapy (RT) [16–20].

ClinicalTrials.gov is the largest clinical trial registry with over 400,000 registered trials and a weekly growth rate of new entries. The registration process and its potential for an analysis of the clinical trials landscape is well described in the literature [21, 22]. A detailed description of registered protocol elements can be found at the ClinicalTrials.gov website [23, 24]. Due to the nature of ClinicalTrials.gov trial submission process, detailed information on past and present clinical trials can be obtained using the ClinicalTrials.gov registry. We focused the current analysis to phase II and III clinical trials for BM, reported in ClinicalTrials.gov in the last decade. The incidence of BM is expected to increase as advancements in modern management of malignant extra-cranial disease have prolonged survival of patients, consequently, the demand for a better management of intracranial disease is increasing. The aim of the current study was to investigate the setup of current clinical trials aimed to improve outcomes in patients with BM through novel therapeutics, improved surveillance, and prevention.

## Material and methods

### Data acquisition

**Fig. 1:**
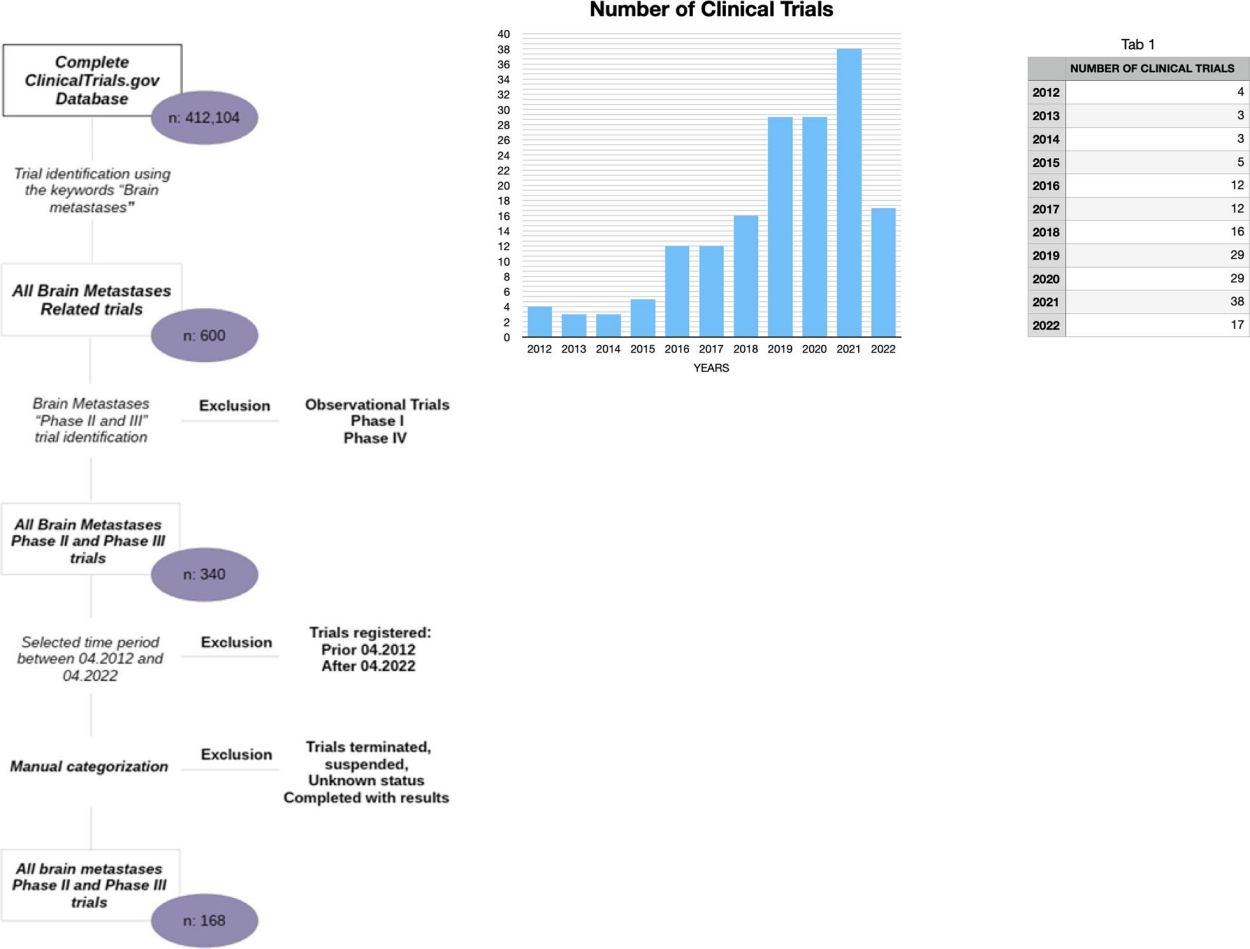
Flow diagram of the trial selection process

The records of all 412,104 clinical trials registered at ClinicalTrials.gov were downloaded on the 15th of April 2022. The following fields were searched for BM and related keywords (brain metastases, tumors metastatic to brain, cerebral metastases): short title, scientific title, conditions, a short summary and detailed description. We analyzed all trials registered during the last ten years (from April 2012 to April 2022). After exclusion of trials that were prematurely closed, completed with results, withdrawn, unknown, suspended, phase I or observational trials, and trials not specific for brain metastases, 168 trials were selected for final analysis. The trial selection process is shown in Fig. 1.

All registered interventions were classified according to their specific role within the trial as part of the standard treatment or the experimental approach. Date of trial registration was considered. Systemic treatments were categorized based on the resources available on the following databases: www.drugbank.ca [25] National Cancer Institute Dictionary of Cancer Terms (www.cancer.gov), pubchem.ncbi.nlm.nih.gov as well as the Scopus database, the PubMed Database, Google Scholar and through a generic internet search (Google search engine).

According to their role in a clinical trial, radiotherapy, surgical procedures, drugs, experimental drugs, and imaging procedures were classified as part of the standard treatment or as an experimental intervention. If the procedure was in the focus of a particular trial, it was considered as an experimental intervention. Similarly, all surgical, as well as RT approaches in BM setting and other interventions that were evaluated within a trial protocol were considered as experimental. Otherwise, all procedures were considered standard treatment. In classifying the characteristics of trials, we used a modified strategy based on the methodology previously described [21].

## Results

A total of 168 trials were selected for the analysis (Additional file 1). Trial design characteristics and a general overview of the trials are shown in Table 1. The number of trials initiated between 2012 and 2017 were 32 (19%), and 136 (81%) those initiated between 2017 and 2022. At the time of analysis, there were 133 phase II trials and 35 phase III trials; amongst them,—13 trials were completed with no published results,—22 trials were active and not recruiting,—107 trials were still actively recruiting participants, and—26 trials were completed and not yet recruiting.

**Table 1:** Trial design characteristics

	n° trials
*Trial start*	
2012–2017	32
2017–2022	136
*Recruitment status*	
Recruiting	107
Not yet recruiting	26
Active, not recruiting	22
Completed	13
*Study phase*	
Phase II	133
Phase III	35
*Funding type*	
Industry	57
NIH	21
All others (university, individuals, organization)	93
*Allocation*	
Randomized	74
Non-randomized	13
N/A	81
*Primary purpose*	
Treatment	158
Diagnostic	5
Prevention	2
Supportive care	2
Other	1
*Primary tumor*	
Lung cancer	67
(NSCLC)	53
(SCLC)	5
(Not-specified)	9
Breast cancer	41
(Her-2 positive)	20
Melanoma	28
(BRAF-mutated)	4
Kidney	2
Primary not-Specified	46
*Interventions*	
Only sistemic treatments (single-associated)	72
Radiotherapy alone	44
Systemic treatment plus radiotherapy	40
Other treatments	1
*Systemic treatments*	
Tyrosine kinase inhibitors	50
Other target therapies	4
Monoclonal antibodies (MABs)	50
MABs immunotherapies target	24
Chemotherapy	20
Other drugs	6
*Radiotherapy*	
Stereotactic radiotherapy (SRS/SRT)	47
Whole-brain RT (WBRT)	27
Hippocampal avoidance WBRT (HA-WBRT)	10
IORT	1
Brachytherapy	1
Technique not specified	10

Most of the trials enrolled patients with BM from lung cancer (67 trials, 39.9%); 53 NSCLC, 5 Small Cell Lung Cancer (SCLC) and 9 not specified. BM from primary breast cancer were evaluated in 41 (24.4%) trials, with 20 trials specifically evaluating HER-2 positive breast cancer patients with BM. Melanoma BM were included in 28 (16.6%) trials, four of them specifically for BRAF mutated melanoma. Forty-six (27.4%) trials included BM from various primary cancers or unspecified histology. The primary purpose was “treatment” in 158 (94%) trials, “diagnostic” in 5 (3%) trials, “supportive” care in 2 (1.2%) trials, “prevention” in 2 (1.2%) trials, and “other” in one (0.6%) trial.

For “treatment” trials, systemic therapies alone given either as single agent or in combination have been evaluated in 73 (46.2%) trials. RT was investigated in 84 trials: 44 trials as exclusive treatment, 40 trials in combination with systemic therapies. Eighty-five trials were single arm with no masking for treatment and 80 trials had two or more treatment arms. Amongst multiple arms trials, 70 were randomized and 10 were non-randomized trials; 75 were with parallel assignment and 5 with sequential assignment. The median patient enrollment size was 70.5 with a maximum size of 601 patients. Fifty-five (32.7%) trials analyzed less than 50 patients in, 63 (37.5%) trials 50–100 patients, and 28 (16.8%) trials from 101 to 200 patients, 11 (6.5%) trials from 201 to 300 patients, and 11 (6.5%) trials more than 300 patients.

### Trials on brain metastases

#### Systemic therapy

**Fig. 2:**
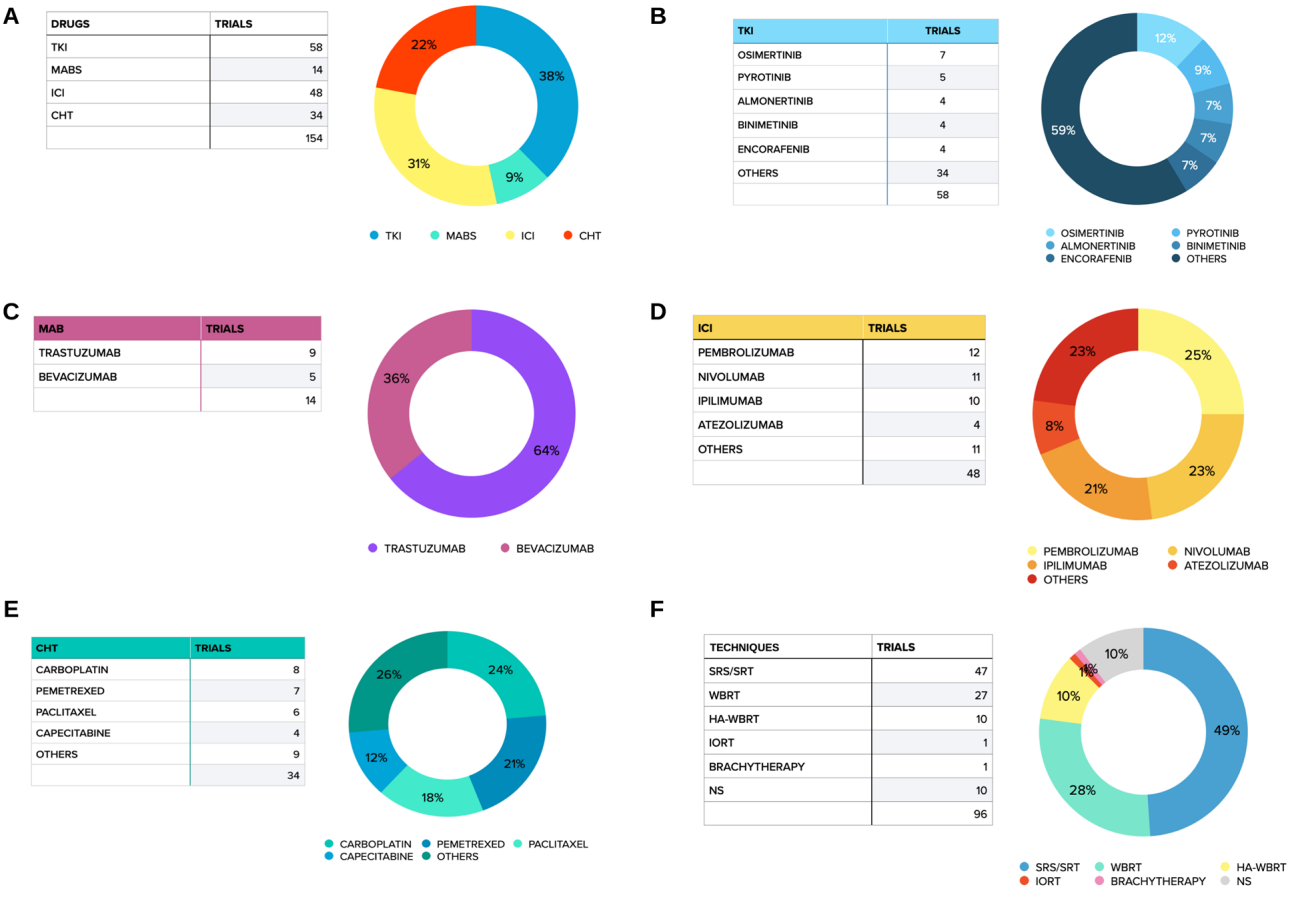
Pie charts of the systemic therapies used

One hundred and fifty-four trials evaluated the use of systemic treatments for patients with BM (Fig. 2A). Fifty-eight trials used TKIs (Fig. 2B), 14 trials used anti-HER2 (9) or anti-VEGF (10) monoclonal antibodies (MAB) (Fig. 2C), 48 trials used immune checkpoint inhibitor (ICI), 34 trials used chemotherapeutic agents (CHT) (Fig. 2D). Sixty-three trials used a single agent and 50 two or more agents; the type and combination of drugs depended on type of primary tumor.

#### Radiotherapy

Ninety-six trials explored the use of RT in patients with BM (Fig. 2E). Amongst them, we identified 63 phase II trials and 33 phase III trials. RT alone was used in 54 trials and in combination with systemic therapy in 42 trials. RT was used as part of standard treatment in 22 (26.2%) trials and as experimental approach in 62 (73.8%) trials. Regarding the radiation technique, SRS and hypofractionated stereotactic radiotherapy (HSRT) were the most used treatments for either intact or surgically resected BMs (47 trials). WBRT was employed in 27 trials, with 10 of them exploring the use of WBRT with hippocampal avoidance (HA-WBRT). Two trials aimed to evaluate the efficacy of preoperative SRS in patients with BM, one trial explored the use of intraoperative radiotherapy (IORT), and one trial the use of brachytherapy. RT technique was not specified In 10 trials.

## Discussion

Trials focusing on BM treatment have been increased over time, with the total number of initiated trials showing a positive trend through the years. Our analysis showed that 32 (19%) trials started between 2012 and 2017 and 136 (81%) started between 2017 and 2022, testifying the availability of new effective treatments for BM, either innovative targeted therapies and immunotherapies or advanced radiation techniques. BM management varies per patient and should involve multidisciplinary discussion as well as patient-centered decision making focusing on maximizing tumor control and minimizing toxicity and improving the quality of life of patient [26]. In this regard, the Diagnosis-Specific Graded Prognostic Assessment (DS-GPA) is a valid prognostic score that might improve shared decision making in clinical practice as well as patient stratification in prospective clinical trials [27].

Current treatment options for patients with BM include surgical resection, WBRT, SRS, and systemic therapy (chemotherapy, targeted therapy, and/or immunotherapy). Surgical resection, usually used for patients with a symptomatic large brain lesion and well controlled systemic disease [28], was present in 4 trials including pre- or post- SRS, intraoperative RT or brachytherapy. No trials are currently exploring new surgical techniques as experimental treatments in the management of BM.

New strategies to reduce neurocognitive decline induced by WBRT, associated or not with simultaneous integrated boost to BMs [29, 30]. a historical mainstay treatment of BM p In our analysis, we found 5 studies aimed to explore WBRT delivered with technique that spare exposure to the hippocampus (NCT04804644 [31], and NCT04277403 [32]), or concurrent delivery of a neuroprotective agent N-Methyl-D-aspartate (NMDA) receptor antagonist memantine (NCT05045950 [33]) or association between hippocampal sparing and memantine use (NCT04801342 [34], NCT03550391 [35]).

SRS and SRT have increasingly used as an alternative to WBRT in patients with BM [20]. Based on randomized studies comparing SRS vs WBRT plus SRS, SRS is the current recommended treatment in patients with a limited number of BM (1–4). Its use has been conditionally recommended up to 10 BM in patients with good performance status [4, 36, 37], by using different approaches, including different monoisocentric techniques [38]. In the present analysis, SRS, given as single-fraction or fractionated schedule (2–5 fractions), is one of the most investigated radiation techniques used in interventional clinical trials for BM as either exclusive treatment or in combination with systemic therapy and surgery. Ongoing trials are exploring the use of preoperative SRS/SRT versus post-operative SRS (NCT04422639 [39], NCT04365374 [40], NCT05124236 [41]), or comparing SRS alone to HA-WBRT in patients with multiple BM (NCT03550391 [35], NCT04277403 [32], NCT03075072 [42]).

Recent data support a role for immunotherapy and targeted systemic therapies as effective treatment for BM [4]. Therefore, it is not surprising that most ongoing trials explore the use of systemic therapies. EGFR TKIs inhibitors are the most used targeted agents in ongoing trials, specifically the third-generation agent osimertinib for its high CNS activity and efficacy in treatment-resistant, EGFR-mutant NSCLC (NCT03769103 [43], NCT02736513 [44], NCT05104281 [45], NCT02971501 [46], NCT03497767 [47], NCT03257124 [48], NCT04233021 [49]). In patients with HER2-postive breast cancer, ongoing trials in BM treatments explore use of two main target therapies, pyrotinib and trastuzumab deruxtecan. Pyrotinib is a novel irreversible EGFR/HER2 dual tyrosine kinase inhibitor used in combination with other drugs (NCT04639271 [50], NCT03691051 [51], NCT03933982 [52]), RT (NCT04582968 [53]) or both (NCT05042791 [54]). Trastuzumab deruxtecan is a HER2-directed antibody and topoisomerase inhibitor conjugate; the DESTINY-Breast01 trial demonstrated that the drug had strong anti-tumor activity in pretreated patients with HER2 positive metastatic breast cancer, especially, those ones with BM [55]. Different ongoing trials are exploring the efficacy of trastuzumab deruxtecan in BM HER-2 positive patients (NCT04752059 [56]; NCT04739761 [57]; NCT04420598 [58]) alone or in combination with other drugs (NCT04538742 [59]).

In patients with melanoma BM, either immunotherapy or BRAF and MEK inhibitors have been associated with high brain control and survival benefit [60–65]. For patients with BRAF-mutant melanoma, four trials are exploring a combination of BRAF and MEK inhibitors agents, including BRAF inhibitor vemurafenib in combination with MEK inhibitor cobimetinib (NCT03430947 [66], NCT02537600 [67]), and BRAF inhibitor encorafenib combined with MEK inhibitor binimetinib (NCT03911869 [68], NCT04511013 [69], NCT03898908 [70]). ICI include large monoclonal antibody-based therapies and small molecule inhibitors that upregulate the immune system and its antitumor activity. Ipilimumab is the most frequent immunotherapy investigated in ongoing trials, given alone or in association with nivolumab. Investigated histologies include melanoma (NCT03903640 [71], NCT02621515 [72]), NSCLC (NCT05012254 [73], NCT02696993 [74]), or mixed histologies (NCT04434560 [75]). Another immunotherapy agent frequently used in ongoing BM trials is the anti-PD-1 monoclonal antibody pembrolizumab. Several trials are currently investigating the use of pembrolizumabgiven alone in patients with BM from multiple histologies (NCT02886585 [76], NCT03563729 [77]), or in combination with ipilimumab for melanoma BM (NCT03873818 [78]), with bevacizumab for melanoma and NSCLC BM (NCT02681549 [79]), with TKI in melanoma and renal cell carcinoma BM (NCT04955743 [80]), in triple-negative breast cancer and NSCLS BM (NCT05064280 [81]), with chemotherapy in NSCLC (NCT04967417 [82]) and triple-negative breast cancer BM (NCT05255666 [83]), and in combination with NovoTTF-200A (Optune) device (NCT04129515 [84]) or SRS in patients with breast cancer BM (NCT03449238 [85]),

## Conclusion

Recent advances both in RT and systemic treatment have created a paradigm shift in the management of BMs. SRS has progressively replaced the use of WBRT in patients with multiple BM for its ability to reduce the risk of neurocognitive decline. Novel targeted therapies and ICI have also revolutionized the systemic management of several tumors showing impressive activity in patients with BM from immunosensitive tumors or harboring druggable mutations. Areas of research for BM being explored in ongoing clinical trials mostly include the evaluation of systemic therapies, given as single agent or two drugs combined, or concurrently to SRS. All these trials intend to address multiple questions on the management of patients with BM, including the recommended upfront treatment for different cancer histologies and the optimal timing between systemic therapies and radiation regarding brain control and neurocognitive outcome and quality of life.

## Supplementary Information

**Additional file 1.** Trials selected for the study.

## Abbreviations

BM: Brain metastases
WBRT: Whole brain radiotherapy
SRS: Stereotactic radiosurgery
TKI: Tyrosine kinase inhibitor
EGFR: Epidermal growth factor receptor
ALK: Anaplastic lymphoma
NSCLC: Non-small-cell lung cancers
HER2: Epidermal growth factor receptor 2
BRAF: V-raf murine sarcoma viral oncogene homolog B1
MEK: Mitogen-activated protein kinase kinase
CTLA-4: Cytotoxic T lymphocyte-associated antigen
MAB: Monoclonal antibodies
ICI: Immune checkpoint inhibitor (ICI)
RT: Radiotherapy
SCLC: Small cell lung cancer
