# Supplementary material for: Current status and perspectives of interventional clinical trials for brain metastases: analysis of ClinicalTrials.gov
Source: Radiat Oncol. 2023 Apr 4;18:62. doi: 10.1186/s13014-023-02243-2 (PMC10074717; doi:10.1186/s13014-023-02243-2)
Supplement: Supplementary file 1 — Additional file 1. Trials selected for the study. [file 13014_2023_2243_MOESM1_ESM.docx]

**Supplementary material 1**

**Trials selected for the study.**

| **TRIAL IDENTIFIER** | **TRIAL STATE** | **DISEASE** | **DESCRIPTION** |
| --- | --- | --- | --- |
| NCT05084092 | Recruiting | Brain | Device: Intraoperative Radiotherapy (IORT) |
| NCT04365374 | Recruiting | Brain | Radiation: Stereotactic Radiation Therapy |
| NCT04222062 | Recruiting | Brain | Drug: Carmustine 7.7Mg Wafer |
| NCT03175432 | Recruiting | Breast | Drug: Atezolizumab\|Biological: Bevacizumab\|Drug: Cobimetinib |
| NCT04789668 | Recruiting | Breast | Drug: Bintrafusp Alfa\|Drug: Pimasertib\|Other: Quality-of-Life Assessment |
| NCT04395339 | Recruiting | Breast | Drug: Monosialotetrahexosyl ganglioside (GM1)\|Drug: Control |
| NCT03449238 | Recruiting | Breast | Drug: Pembrolizumab |
| NCT03190967 | Recruiting | Breast | Drug: T-DM1\|Drug: TMZ |
| NCT04752059 | Recruiting | Breast | Drug: Trastuzumab deruxtecan |
| NCT04739761 | Recruiting | Breast | Drug: Trastuzumab Deruxtecan |
| NCT04538742 | Recruiting | Breast | Drug: Trastuzumab deruxtecan\|Durvalumab\|Paclitaxel\|Pertuzumab\|Tucatinib |
| NCT03765983 | Recruiting | Breast | Drug: Trastuzumab\|Drug: GDC-0084 |
| NCT05041842 | Recruiting | Breast | Drug: Tucatinib\|Drug: Pertuzumab\|Drug: Trastuzumab\|Drug: Hormone therapy |
| NCT04582968 | Recruiting | Breast | Other: Pyrotinib Plus Capecitabine combined with brain radiotherapy |
| NCT04923542 | Recruiting | Breast | Radiation: SRS \|Drug: Abemaciclib\|Drug: Endocrine therapy |
| NCT05255666 | Not yet recruiting | Breast | Drug: Pembrolizumab\|Drug: Liposomal Irinotecan |
| NCT04639271 | Not yet recruiting | Breast | Drug: Pyrotinib Plus And\|Drug: Trastuzumab\|Drug: Abraxane |
| NCT04760431 | Not yet recruiting | Breast | Drug: Trastuzumab\|Drug: Taxanes\|Pertuzumab\|Tyrosine kinase inhibitor |
| NCT05323955 | Not yet recruiting | Breast | Drug: Trastuzumab\|Trastuzumab Emtansine (T-DM1)\|Pertuzumab\|Tucatinib |
| NCT02048059 | Completed | Breast | Drug: ANG1005 |
| NCT04420598 | Completed | Breast | Drug: Trastuzumab deruxtecan |
| NCT03483012 | Active, not recruiting | Breast | Drug: Atezolizumab\|Procedure: Stereotactic radiosurgery (SRS) |
| NCT02185352 | Active, not recruiting | Breast | Drug: BEEP regimen |
| NCT03691051 | Active, not recruiting | Breast | Drug: Pyrotinib plus Capecitabine |
| NCT02898727 | Active, not recruiting | Breast | Other: Local Therapy |
| NCT05042791 | Recruiting | Breast | Combination Product: radiation combined with pyrotinib and capecitabine |
| NCT03331601 | Recruiting | Breast | Drug: 68GaNOTA-Anti-HER2 VHH1 |
| NCT04158947 | Recruiting | Breast | Drug: Afatinib\|Drug: T-DM1 |
| NCT03328884 | Recruiting | Breast | Drug: Irinotecan Hydrochloride |
| NCT03933982 | Recruiting | Breast | Drug: Pyrotinib Plus Vinorelbine |
| NCT04061408 | Recruiting | Breast | Radiation: Fractionated stereotactic radiotherapy(FSRT) |
| NCT05144867 | Recruiting | Breast | Radiation: Stereotactic Radiosurgery |
| NCT04791384 | Not yet recruiting | Breast | Drug: Abemaciclib\|Drug: Elacestrant |
| NCT04965064 | Not yet recruiting | Breast | Drug: Capecitabine and Neratinib. |
| NCT04711824 | Not yet recruiting | Breast | Drug: Olaparib\|Radiation: SR \| Drug: Durvalumab |
| NCT05305365 | Not yet recruiting | Breast | Drug: QBS72S |
| NCT02595905 | Active, not recruiting | Breast | Drug: Cisplatin\|Other: Placebo Administration\|Drug: Veliparib |
| NCT04647916 | Recruiting | Breast - Brain | Biological: Sacituzumab Govitecan |
| NCT05018702 | Recruiting | Brest | Drug: ARX788 |
| NCT03951142 | Recruiting | Glioblastoma | Drug: Losartan |
| NCT05326425 | Recruiting | Lung | Drug: lazertinib(YH25448) |
| NCT04567251 | Recruiting | Lung | Drug: NamzaricÂ®\|Drug: Placebo |
| NCT04964960 | Recruiting | Lung | Drug: Pembrolizumab - Paclitaxel- Pemetrexed-Carboplatin |
| NCT04808752 | Not yet recruiting | Lung | Drug: Almonertinib |
| NCT02736513 | Active, not recruiting | Lung | Drug: AZD9291 |
| NCT04634110 | Active, not recruiting | Lung | Drug: Brigatinib |
| NCT02768337 | Recruiting | Lung - Breast | Drug: Afatinib\|Radiation: 2 Gy \|Radiation: 4 Gy |
| NCT04700072 | Recruiting | Melanoma | Biological: Pembrolizumab\| Pembrolizumab/Quavonlimab\|Drug: Lenvatinib |
| NCT03903640 | Recruiting | Melanoma | Device: Optune\|Biological: Nivolumab\|Biological: Ipilimumab |
| NCT04021420 | Recruiting | Melanoma | Device: SONOCLOUD\|Drug: Nivolumab Injection |
| NCT03898908 | Recruiting | Melanoma | Drug: encorafenib\|binimetinib\|Radiation: WBRT - Radiation: SR |
| NCT04674683 | Recruiting | Melanoma | Drug: HBI-8000 in combination with nivolumab |
| NCT03340129 | Recruiting | Melanoma | Drug: Ipilimumab\|Drug: Nivolumab\|Radiation: SR |
| NCT03563729 | Recruiting | Melanoma | Pembro-Ipili-Nivo-Encorafenib-Binimetinib-Dabrafenib-Trametinib |
| NCT02681549 | Recruiting | Melanoma | Drug: Pembrolizumab plus Bevacizumab |
| NCT03741673 | Recruiting | Melanoma | Other: Quality-of-Life Assessment\| Questionnaire Administration\|Radiation: SRS |
| NCT02537600 | Completed | Melanoma | Drug: Cobimetinib + Vemurafenib combination treatment |
| NCT02662725 | Completed | Melanoma | Drug: IPILIMUMAB\|Radiation: Stereotactic radiosurgery |
| NCT03430947 | Active, not recruiting | Melanoma | Drug: Vemurafenib\|Drug: Cobimetinib |
| NCT01644591 | Active, not recruiting | Melanoma | Other: Quality-of-Life Assessment\|Questionnaire Administration\|Radiation: SRS |
| NCT04129515 | Recruiting | Melanoma | Device: NovoTTF-200A\|Drug: Pembrolizumab |
| NCT04511013 | Recruiting | Melanoma | Drug: Binimetinib\|Encorafenib\|Biological: Ipilimumab\|Nivolumab |
| NCT04899921 | Recruiting | Melanoma | Drug: Ipilimumab\|Drug: Nivolumab\|Drug: Troriluzole\|Drug: Placebo |
| NCT02374242 | Active, not recruiting | Melanoma | Drug: Nivolumab\|Drug: Ipilimumab |
| NCT03873818 | Recruiting | Melanoma - Brain | Biological: Ipilimumab\|Biological: Pembrolizumab |
| NCT04899908 | Recruiting | Melanoma- Breast - Lung | Radiation: SRS \|Drug: AGuIX gadolinium-based nanoparticles |
| NCT04955743 | Recruiting | Melanoma- Renal | Drug: Pembrolizumab\|Drug: Lenvatinib |
| NCT03994796 | Recruiting | ns | Drug: Abemaciclib\|Drug: PI3K Inhibitor paxalisib\|Drug: Entrectinib |
| NCT03818386 | Recruiting | ns | Drug: AGuIXÂ®\|Radiation: Whole Brain Radiation Therapy |
| NCT03608020 | Recruiting | ns | Drug: BMX-001\|Radiation: Whole Brain Radiation Therapy |
| NCT04461418 | Recruiting | ns | Drug: Glucocorticoid therapy |
| NCT03550391 | Recruiting | ns | Drug: Memantine\|Radiation: HA-WBRT - SRS |
| NCT02896335 | Recruiting | ns | Drug: Palbociclib |
| NCT05064280 | Recruiting | ns | Drug: pembrolizumab\|Drug: Lenvatinib |
| NCT02886585 | Recruiting | ns | Drug: Pembrolizumab\|Procedure: SR |
| NCT03778541 | Recruiting | ns | Drug: Temozolomide\|Radiation: hypofractionated radiotherapy |
| NCT05222620 | Recruiting | ns | Other: Questionnaire Administration\|Radiation: Stereotactic Radiosurgery |
| NCT04474925 | Recruiting | ns | Procedure: Brain Surgery\|Radiation: Stereotactic Radiosurgery |
| NCT03184038 | Recruiting | ns | Procedure: Cognitive Assessment\|Radiation: SRS |
| NCT03750227 | Recruiting | ns | Procedure: Conventional Surgery\|Questionnaire Administration\|Radiation: SRS |
| NCT04690348 | Recruiting | ns | Procedure: Craniotomy\|Radiation: Cesium-131 brachytherapy |
| NCT04503772 | Recruiting | ns | Procedure: Preoperative SRS |
| NCT02747303 | Recruiting | ns | Procedure: Stereotactic Radiosurgery |
| NCT04343157 | Recruiting | ns | Radiation: Cognitive Sparing Brain Stereotactic Radiosurgery (SRS) |
| NCT04801342 | Recruiting | ns | Radiation: HA-WBRT \|Drug: Memantine Hydrochloride |
| NCT03696680 | Recruiting | ns | Radiation: FSRT Stereotactic radiation therapy |
| NCT04452084 | Recruiting | ns | Radiation: HA-WBRT\|Radiation: HA-SIB-WBRT |
| NCT04277403 | Recruiting | ns | Radiation: HA-WBRT\|Radiation: SRS |
| NCT02504788 | Recruiting | ns | Radiation: hippocampal-sparing WBRT |
| NCT05267587 | Recruiting | ns | Radiation: Hypofractionated Stereotactic Radiosurgery |
| NCT04829019 | Recruiting | ns | Radiation: Osimertinib and whole-brain irradiation\|Drug: Osimertinib |
| NCT04427228 | Recruiting | ns | Radiation: Radiosurgery Single Treatment\|Radiation: Radiosurgery |
| NCT04114981 | Recruiting | ns | Radiation: Single Fraction SRS \|Radiation: Fractionated SRS \|Questionnaire Adm. |
| NCT03028337 | Recruiting | ns | Radiation: Spine Radiosurgery\|Behavioral: Questionnaires |
| NCT04422639 | Recruiting | ns | Radiation: SRS/SRT |
| NCT03508752 | Recruiting | ns | Radiation: Stereotactic Radiosurgery |
| NCT03075072 | Recruiting | ns | Radiation: Whole brain radiation\|Radiation: Stereotactic radiation (SRS) |
| NCT04197297 | Not yet recruiting | ns | Diagnostic Test: CT and MRI Scans |
| NCT04834388 | Not yet recruiting | ns | Drug: Anakinra |
| NCT05177185 | Not yet recruiting | ns | Radiation: Hippocampal-sparing\|Radiation: Stereotactic radiosurgery |
| NCT03697343 | Not yet recruiting | ns | Radiation: Radiosurgery\|Radiation: Fractionated stereotactic radiotherapy |
| NCT05050929 | Not yet recruiting | ns | Radiation: Targeting All Brain Metastases |
| NCT03668847 | Completed | ns | Drug: DM-CHOC-PEN |
| NCT03911869 | Completed | ns | Drug: encorafenib\|Drug: binimetinib |
| NCT02104193 | Completed | ns | Drug: simvastatin in addition to rt\|Radiation: radiation therapy |
| NCT02866981 | Completed | ns | Other: Observation |
| NCT02147028 | Completed | ns | Radiation: HA-WBRT |
| NCT01942980 | Completed | ns | Radiation: Radiation therapy Brain metastasis in breast cancer |
| NCT04338867 | Completed | ns | Radiation: Whole-brain radiotherapy |
| NCT04410367 | Active, not recruiting | ns | Drug: 18F fluciclovine |
| NCT04410133 | Active, not recruiting | ns | Drug: 18F fluciclovine |
| NCT01592968 | Active, not recruiting | ns | Other: Cognitive Assessment\|Radiation: SRS \|Radiation: WBRT |
| NCT02798029 | Active, not recruiting | ns | Radiation: Frameless Fractionated Stereotactic Radiation Therapy |
| NCT03789149 | Active, not recruiting | ns | Radiation: Intraoperative Radiotherapy |
| NCT03285932 | Active, not recruiting | ns | Radiation: SRS\|Radiation: WBRT |
| NCT04946019 | Recruiting | NSCLC | Combination Product: Unity-based MR-Linac guided FSRT |
| NCT02831959 | Recruiting | NSCLC | Device: NovoTTF-200M device\|Other: Best Standard of Care |
| NCT02521051 | Recruiting | NSCLC | Drug: Alectinib\|Drug: Bevacizumab |
| NCT04905550 | Recruiting | NSCLC | Drug: Almonertinib\|Radiation: SRT or SRS or WBRT |
| NCT04978753 | Recruiting | NSCLC | Drug: Anlotinib |
| NCT04147728 | Recruiting | NSCLC | Drug: Anlotinib\|Radiation: Stereotactic Radiosurgery |
| NCT04675008 | Recruiting | NSCLC | Drug: Dacomitinib |
| NCT01951469 | Recruiting | NSCLC | Drug: Gefitinib and Pemetrexed/platinum\|Drug: Gefitinib mono-therapy |
| NCT02726568 | Recruiting | NSCLC | Drug: Icotinib\|Radiation: SRS |
| NCT04058704 | Recruiting | NSCLC | Drug: Icotinib\|Radiation: SRS/WBRT/HA-WBRT/SMART |
| NCT04291092 | Recruiting | NSCLC | Drug: Immunotherapy\|Radiation: WBRT\|Drug: Chemotherapy |
| NCT05012254 | Recruiting | NSCLC | Drug: Ipilimumab\|Nivolumab\| Carboplatin \| Cisplatin \| Paclitaxel Pemetrexed |
| NCT04824079 | Recruiting | NSCLC | Drug: Keynatinib |
| NCT05045950 | Recruiting | NSCLC | Drug: Memantine \|Radiation: WBRT |
| NCT04233021 | Recruiting | NSCLC | Drug: Osimertinib |
| NCT05104281 | Recruiting | NSCLC | Drug: osimertinib oral and bevazizumab intravenously |
| NCT03497767 | Recruiting | NSCLC | Drug: Osimertinib\|Radiation: Stereotactic Radiosurgery (SRS) |
| NCT03769103 | Recruiting | NSCLC | Drug: Osimertinib\|Radiation: Stereotactic radiotherapy |
| NCT04213170 | Recruiting | NSCLC | Drug: sintilimab |
| NCT05207904 | Recruiting | NSCLC | Drug: Tislelizumab, paclitaxel, Carboplatin |
| NCT04415320 | Recruiting | NSCLC | Drug: X-396(Ensartinib) |
| NCT02696993 | Recruiting | NSCLC | Ipilimumab\|Nivolumab\|Radiation: SRS \|Radiation: WBRT |
| NCT02448992 | Recruiting | NSCLC | Radiation: hippocampal-sparing WBRT |
| NCT05236946 | Recruiting | NSCLC | Radiation: SRS/WBRT\|Drug: Tyrosine kinase inhibitor |
| NCT04643847 | Not yet recruiting | NSCLC | Drug: Almonertinib |
| NCT04870190 | Not yet recruiting | NSCLC | Drug: Almonertinib\|Drug: Osimertinib |
| NCT05180422 | Not yet recruiting | NSCLC | Drug: AMG 510\|Drug: MVASI |
| NCT04768075 | Not yet recruiting | NSCLC | Drug: Camrelizumab Cisplatin Carboplatin\|Pemetrexed\|Paclitaxel |
| NCT04339829 | Not yet recruiting | NSCLC | Drug: Dacomitinib |
| NCT04193007 | Not yet recruiting | NSCLC | Drug: molecular targeted therapies\|Radiation: Brain Radiotherapy |
| NCT04967417 | Not yet recruiting | NSCLC | Drug: Pemetrexed, Carbo, Pembro\|Paclitaxel, Carbo, Pembro |
| NCT03732482 | Not yet recruiting | NSCLC | Drug: Temozolomide capsules\|Device: radiotherapy |
| NCT05146219 | Not yet recruiting | NSCLC | Drug: TY-9591 Tablets |
| NCT04889066 | Not yet recruiting | NSCLC | Radiation: Stereotactic Radiation Therapy\|Drug: Durvalumab |
| NCT01887795 | Completed | NSCLC | Drug: Erlotinib\|Drug: WBRT |
| NCT01724801 | Completed | NSCLC | Radiation: whole brain radiation(WBI)\|Drug: Icotinib |
| NCT03526900 | Active, not recruiting | NSCLC | Drug: Atezolizumab |
| NCT03257124 | Active, not recruiting | NSCLC | Drug: AZD9291 |
| NCT03046992 | Active, not recruiting | NSCLC | Drug: YH25448 |
| NCT04211090 | Recruiting | NSCLC | Drug: Camrelizumab\|Drug: Pemetrexed\|Drug: Carboplatin |
| NCT04610684 | Recruiting | NSCLC | Drug: Carboplatin\|Drug: Etoposide\|Drug: Atezolizumab |
| NCT02714010 | Recruiting | NSCLC | Drug: EGFR-TKI\|Radiation: whole brain radiotherapy |
| NCT04507217 | Not yet recruiting | NSCLC | Drug: Tislelizumab, Carboplatin, Pemetrexed |
| NCT03653546 | Active, not recruiting | NSCLC | Drug: AZD3759\|Drug: Erlotinib\|Drug: Gefitinib |
| NCT02971501 | Active, not recruiting | NSCLC - Brain | Biological: Bevacizumab\|Drug: Osimertinib |
| NCT04588246 | Recruiting | NSCLC - Breast | Drug: Memantine\|Radiation: SR \|Radiation: WBRT |
| NCT04755920 | Not yet recruiting | Rectal | Drug: SGM-101 |
| NCT03967522 | Recruiting | Rectum | Drug: Cabozantinib |
| NCT02978404 | Active, not recruiting | Renal- NSCLC - SCLC | Drug: Nivolumab\|Radiation: Radiosurgery |
| NCT04804644 | Recruiting | SCLC | Drug: Memantine \|Radiation: SR \|Radiation: WBRT |
| NCT03995667 | Recruiting | SCLC | Other: Questionnaire Administration\|Device: Tumor Treating Fields Therapy |
| NCT04516070 | Recruiting | SCLC | Other: Questionnaire Administration\|Radiation: Stereotactic Radiosurgery |
| NCT04535739 | Recruiting | SCLC | Radiation: prophylactic cranial irradiation |
